# WebPDF: a browser-based software application for calculating X-ray pair distribution function

**DOI:** 10.1007/s44211-026-00917-x

**Published:** 2026-05-01

**Authors:** Hiroki Yamada, Takuto Fukuoka, Kei Watanabe, Seiya Shimono, Yuki Sada, Kengo Nakada, Toshiyuki Matsunaga, Yoshiharu Uchimoto, Koji Ohara

**Affiliations:** 1 Japan Synchrotron Radiation Research Institute/SPring-8, Kouto 1-1-1, Sayo-cho, Sayo-gun, Hyogo 679-5198 Japan; 2 Faculty of Materials for Energy, Shimane University, 1060 Nishi-Kawatsu-cho, Matsue, Shimane 690-8504 Japan; 3 SPring-8 Service Co., Ltd., Kouto 1-20-5, Shingu-cho, Tatsuno, Hyogo 679-5165 Japan; 4 Graduate School of Human and Environment Studies, Kyoto University, Kyoto, 606-8501 Japan; 5 Institute for Materials Research, Tohoku University, Tohoku University Satellite Office, Nakamozu Campus, Osaka Metropolitan University 1-1 Gakuen-cho, Naka-ku, Sakai, Osaka, 599-8531 Japan; 6 Co-Creation Institute for Advanced Materials, Shimane University, 1060 Nishi-Kawatsu-cho, Shimane, 690-8504 Japan

**Keywords:** X-ray diffraction, X-ray total scattering, X-ray pair distribution function, Software

## Abstract

Pair distribution function (PDF) analysis based on X-ray total scattering is a powerful technique for elucidating local and medium-range structure in crystalline, nanocrystalline, and amorphous materials. Several software packages—such as GudrunX, PDFgetX2, and PDFgetX3—along with facility-specific programs developed at synchrotron sites, are widely used for this purpose. However, these tools typically require installation and environment-dependent configuration, which can introduce compatibility issues across operating systems and create conflicts with pre-installed software. To overcome these limitations, we developed a browser-based PDF analysis application implemented using Pyodide and PyScript, allowing it to run entirely in modern web browsers without additional installation. The software provides key functionalities, including Fourier transformation from *S*(*Q*) to *G*(*r*), calculation of *g*(*r*), back-Fourier transformation with cutoff handling, and sequential data processing. Demonstrations on representative samples confirm consistency with established programs. The software supports both synchrotron data and laboratory-based measurements using Ag radiation sources, underscoring its broad applicability. This tool enables environment-independent and consistent analyses, significantly enhancing the accessibility of X-ray total-scattering and PDF techniques.

**Graphical abstract:**

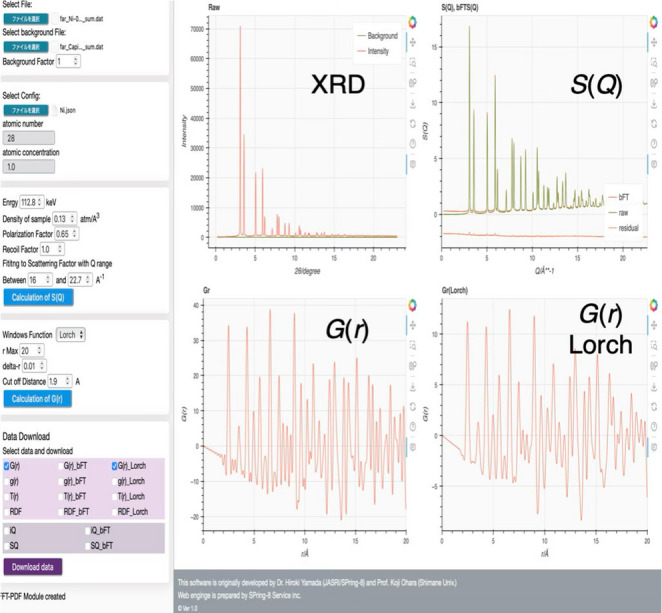

**Supplementary Information:**

The online version contains supplementary material available at 10.1007/s44211-026-00917-x.

## Introduction

Pair distribution function (PDF) analysis derived from X-ray total scattering has become a critical method for probing atomic arrangements that extend beyond the long-range periodicity accessible to conventional crystallography [1]. In crystalline materials, Bragg diffraction captures the averaged periodic structure, while diffuse and total scattering encode essential information on local and medium-range order [2]. Fourier transformation of the total-scattering signal into real space yields a quantitative distribution of interatomic distances, enabling detailed characterization of disordered, nanocrystalline, and amorphous systems [3]. This approach has been widely applied to materials such as oxide and chalcogenide glasses, metallic glasses, catalysts, and battery electrodes, providing key insights into short-range correlations and defect-induced structural distortions. For example, X-ray PDF analysis combined with complementary techniques such as neutron scattering and computer simulations was used to investigate the local structure of amorphous Li₃PS₄ glassy solid electrolytes and reveal structural features that enhance Li-ion diffusion [4].

However, although PDF analysis is a powerful tool for investigating materials lacking long-range periodicity, it is not an element-selective method. Unlike techniques such as X-ray absorption fine structure (XAFS) [5], PDF analysis provides structural information averaged over all atomic species present. Therefore, particular care is required when interpreting contributions from minor or trace elements. In addition, reliable PDF extraction requires high-quality total-scattering data over a sufficiently wide *Q* range together with careful corrections and normalization procedures [6]. These requirements highlight the importance of robust experimental conditions, reliable data-reduction workflows, and sufficiently wide-*Q*-range data for accurate PDF analysis.

Significant progress in synchrotron radiation facilities has accelerated the development and application of PDF analysis. Dedicated beamlines for total scattering and PDF measurements are now operating at major synchrotron sources worldwide, including BL04B2 [7, 8] and BL08W [9] at SPring-8 (Japan), 11-ID-B [10] at the Advanced Photon Source (USA), ID22[11] at ESRF (France), P02.1 [12] at PETRA III (Germany), and I15-1 [13] at Diamond Light Source (UK). These beamlines provide high-flux, high-energy X-rays (*E* > 60 keV) and broad *Q*_max_ coverage (> 25 Å^−1^), enabling accurate determination of local structural correlations across an extended real-space range. The integration of high-energy X-rays with fast two-dimensional detectors has also improved measurement throughput [14] and enabled in situ and time-resolved experiments under controlled thermal, mechanical, and chemical environments [10].

Moreover, recent advances in laboratory instrumentation have further expanded the reach of PDF analysis. Modern X-ray diffractometers equipped with Ag or Mo Kα sources such as Rigaku SmartLab diffractometer (Rigaku Corporation, Japan), now support total-scattering measurements suitable for PDF extraction [15, 16]. Such systems, typically equipped with dedicated optics and one-dimensional semiconductor detectors, enable the acquisition of reliable PDFs up to *Q*_max_ values of approximately 20 Å^−1^. This capability has substantially broadened the accessibility of PDF analysis, making it practical not only at large-scale facilities but also in university and industrial laboratories. Consequently, PDF analysis is emerging as a versatile structural probe that bridges conventional diffraction, spectroscopy, and computational modeling [17].

To accurately compute X-ray PDF patterns, several software packages have been developed to implement the required data-reduction steps [18], including PDFgetX2 [19], PDFgetX3 [20], GudrunX [16] and DAWN [21]. These programs have played a key role in establishing standardized workflows for total-scattering and PDF analysis. However, most still require local installation and environment setup, often involving dependencies such as specific Python versions and associated libraries. For industrial and general users without programming expertise—and even for researchers—installing and maintaining these packages can be challenging, particularly in restricted computing environments. Despite being well-documented and versatile, they continue to face issues related to cross-platform compatibility and version control across Windows, macOS, and Linux.

At SPring-8 in Japan, facility-specific programs developed at the beamlines, which is built on the commercial Igor Pro framework, are usually used to calculate X-ray PDF patterns [22]. Although reliable within the facility, these programs are highly dependent on the operating environment and are not easily distributed due to licensing limitations, rigid configurations, and requirements for manually prepared parameter files. These constraints limit reproducibility across institutions and hinder remote or collaborative analysis.

To overcome these challenges and expand access to PDF analysis, we developed WebPDF, a browser-based platform capable of performing the major steps of total-scattering data processing without local installation. Unlike conventional software that depends on specific operating systems or preconfigured computational environments, WebPDF employs WebAssembly—designed to provide near-native execution speed within web browsers—together with in-browser Python execution (e.g., Pyodide) to perform computationally demanding data-reduction procedures. Owing to the high-performance execution model of WebAssembly, browser-based scientific computing has become practically feasible. In this study, we implement this approach for X-ray PDF analysis and validate its practical utility.

Operating entirely within a web environment, WebPDF offers a portable and reproducible analysis platform that is suitable for both synchrotron and laboratory measurements. Importantly, this approach is not limited to PDF analysis and can be extended to other analysis methods, such as Rietveld refinement [23, 24] and XAFS data processing [25], providing a practical basis for future development of web-based analytical software.

## Calculation of X-ray PDF patterns

In X-ray total-scattering experiments, the measured intensity $I(Q)$ consists of coherent and incoherent components:

$$I\left(Q\right)={I}_{\mathrm{coh}}\left(Q\right)+{I}_{\mathrm{incoh}}\left(Q\right),$$

where $Q=4\pi \mathrm{sin}\theta /\lambda$ is the magnitude of the scattering vector. The coherent term ${I}_{\mathrm{coh}}(Q)$ carries structural information and is given by

$${I}_{\mathrm{coh}}\left(Q\right)=\sum_{i}\sum_{j}{c}_{i}{c}_{j}{f}_{i}\left(Q\right){f}_{j}\left(Q\right)\frac{\mathrm{sin}\left(Q{r}_{ij}\right)}{Q{r}_{ij}},$$

where ${c}_{i}$ and ${f}_{i}(Q)$ are the atomic fraction and atomic form factor [19] of the $i$-th element, respectively, and ${r}_{ij}$ is the interatomic distance between atoms $i$ and $j$. The incoherent term ${I}_{\mathrm{incoh}}(Q)$ arises primarily from Compton scattering [26], which increases with scattering angle and must be corrected to isolate the structural signal. In practice, background subtraction, polarization, absorption, and Compton corrections are required to obtain the coherent intensity. The structure factor [27, 28] is defined as

$$S\left(Q\right)=\frac{{I}_{\mathrm{coh}}\left(Q\right)-\langle f(Q{)}^{2}\rangle }{\langle f(Q{)\rangle }^{2}}+1,$$

where $\langle f(Q{)}^{2}\rangle =\sum_{i}{c}_{i}{f}_{i}(Q{)}^{2}$ and $\langle f(Q)\rangle =\sum_{i}{c}_{i}{f}_{i}(Q)$ represent the mean-square and mean atomic form factors, respectively. Ideally, $S(Q)$ oscillates around unity; deviations indicate incomplete background removal or improper normalization. The reduced structure function is then defined as

$$F\left(Q\right)=Q\left[S\left(Q\right)-1\right].$$

The reduced pair distribution function $G(r)$ is obtained by a sine Fourier transform of $F(Q)$ over the experimental $Q$-range:

$$G\left(r\right)=\frac{2}{\pi }{\int }_{{Q}_{\mathrm{min}}}^{{Q}_{\mathrm{max}}}F\left(Q\right)\mathrm{sin}\left(Qr\right)\hspace{0.17em}\mathrm{d}Q.$$

$G(r)$ describes the distribution of interatomic distances (units of Å^−2^). Because the integration range is finite, truncation at ${Q}_{\mathrm{max}}$ introduces oscillatory artifacts known as termination ripples. The atomic pair correlation function is given by

$$g\left(r\right)=1+\frac{G\left(r\right)}{4\pi r{\rho }_{0}}.$$

where ${\rho }_{0}$ is the atomic number density. The lower real-space limit (*r*_min_) is determined from the behavior of $g(r)$, typically by excluding the nonphysical region close to the origin and restricting the structural range of interest. After applying the cutting procedure at low *r*, the modified function becomes

$${G}_{\mathrm{cut}}\left(r\right)=4\pi r{\rho }_{0}\left[{g}_{\mathrm{cut}}\left(r\right)-1\right].$$

To check consistency between real- and reciprocal-space data, a back Fourier transform is performed to reconstruct the structure function ${S}_{\mathrm{bft}}(Q)$. This procedure allows direct assessment of the influence of cutoff parameters on the reciprocal-space profile and helps ensure agreement between $S(Q)$ and $G(r)$. The updated reduced structure function is then defined as

$${F}_{\mathrm{new}}\left(Q\right)=Q\left[{S}_{\mathrm{bft}}\left(Q\right)-1\right].$$

To suppress termination ripples and improve smoothness, a window function $W(Q)$, typically the Lorch function, is applied to ${F}_{\mathrm{new}}(Q)$ before the final Fourier transform:

$${G}_{\mathrm{Lorch}}\left(r\right)=\frac{2}{\pi }{\int }_{{Q}_{\mathrm{min}}}^{{Q}_{\mathrm{max}}}{F}_{\mathrm{new}}\left(Q\right)W\left(Q\right)\mathrm{sin}\left(Qr\right)\hspace{0.17em}\mathrm{d}Q.$$

The achievable real-space resolution and maximum observable distance depend on the reciprocal-space sampling conditions:

$$\Delta r=\frac{\pi }{{Q}_{\mathrm{max}}}, {r}_{\mathrm{max}}=\frac{\pi }{\Delta Q}.$$

Appropriate selection of ${Q}_{\mathrm{max}}$ and the sampling interval $\Delta Q$ is therefore essential for balancing real-space resolution with the accessible structural range. Poor choices can lead to aliasing or excessive smoothing in $G(r)$, making interactive optimization within the software interface particularly valuable.

## Program architecture and availability

The WebPDF software is implemented using WebAssembly and PyScript, enabling Python-based numerical routines to run directly within standard web browsers (e.g. Microsoft Edge, Google Chrome and Safari). Its computational core leverages established scientific libraries, including NumPy and SciPy, ensuring accuracy and efficiency in Fourier transformation, normalization, and background-subtraction operations. Interactive, high-quality visualizations of scattering and PDF profiles are generated using the Bokeh library.

By adopting this architecture, the software eliminates the need for local installation of external packages and provides performance comparable to conventional desktop applications. The execution environment is independent of the operating system (e.g., Windows, Linux, macOS) and processor architecture (e.g., × 86–64, ARM64), thereby avoiding common compatibility issues. The combination of WebAssembly and PyScript enables optimized numerical performance while maintaining a responsive user interface.

The complete source code, along with utility software required for preprocessing, is openly available on GitHub. This ensures transparency of implementation and facilitates validation, reproducibility, and community-driven development.

## Input files required for WebPDF software

For calculating PDFs using the WebPDF software, three types of input files are typically required. Example files for these three formats are available on the WebPDF website (https://yamada-hiroki.com/software.html). The first two are the X-ray total-scattering patterns of the sample and the corresponding background cell, each provided in a two-column format containing scattering angle (2θ) and intensity. Although the background file is technically optional, its use is strongly recommended because background subtraction is essential for accurate PDF determination. The software accepts space-, comma-, and tab-delimited text files, ensuring compatibility with common diffractometer output formats such as generic ASCII outputs widely used in laboratory X-ray diffractometers and synchrotron beamlines.

The third input file is a JavaScript Object Notation (JSON) configuration file that defines the parameters required for the calculation. These include the incident X-ray energy, elemental concentrations, and other quantities necessary for normalization and Fourier transformation. A complete list of parameters is provided in Table 1. Because WebPDF operates entirely within a web browser, it avoids complications associated with software installation or conflicts with existing computing environments. All calculations are performed locally on the user’s device, ensuring that no experimental data or sample-related information is transmitted to external servers.

**Table 1:** Parameters defined in the JSON input file for WebPDF (example: SiO_2_ glass), which were used in the analysis shown in Fig. 1

Parameter name	Description	Value (example of SiO_2_ glass)
Atomic number density	Number density of atoms (Å^−3^)	0.066
Polarization factor	Correction factor for polarization	0.52
Recoil factor	Correction factor for atomic recoil	1.0
*Q*_min_	Minimum momentum transfer for Fourier transform (Å^−1^)	1.0
*Q*_max_	Maximum momentum transfer for Fourier transform (Å^−1^)	22.0
*r*_max_	Maximum real-space distance for *G*(*r*) calculation (Å)	10.0
Δ*r*	Step-size of *G*(*r*) (Å)	0.01
Cutoff distance	Lower cutoff distance applied to *G*(*r*) (Å)	1.0
X-ray energy	Incident X-ray energy (keV)	22.148
Atomic numbers	Atomic numbers of constituent elements	14 (Si), 8 (O)
Atomic concentrations	Relative atomic concentrations	1 (Si), 2 (O)

## X-ray total-scattering measurements

Laboratory-based X-ray total-scattering measurements were performed using a Rigaku SmartLab diffractometer (Rigaku Corporation, Japan) equipped with a sealed-tube Ag Kα radiation source (22.148 keV, 40 kV, 200 mA). A silica (SiO_2_) glass rod was measured in Debye–Scherrer transmission geometry without a capillary. Diffracted intensities were collected using a one-dimensional D/teX Ultra250-HE semiconductor detector. A background profile was acquired separately under identical conditions, with no sample mounted on the spinner stage. Data were collected over a 2θ range of 3.0–157° with a step size of 0.01° and the exposure time for each measurement was 20 h.

Synchrotron X-ray total-scattering data were collected at the BL04B2 beamline of SPring-8. Powder samples of Ni and KNbO_3_ (φ = 0.8 mm) were packed into borosilicate capillaries and measured with an exposure time of 10 min. Monochromatic X-rays with an energy of 112.8 keV (λ = 0.110 Å) were produced using a Si (511) single-crystal monochromator. Scattered intensities were recorded with a CdTe LAMBDA 750 k area detector (X-Spectrum GmbH) placed at a sample-to-detector distance of ~ 510 mm. The two-dimensional diffraction images obtained at BL04B2 were azimuthally integrated into one-dimensional 2θ–intensity profiles using the pyFAI software package, whereas the SmartLab data were directly exported in 2θ–intensity format.

## Typical results obtained by WebPDF software

**Fig. 1:**
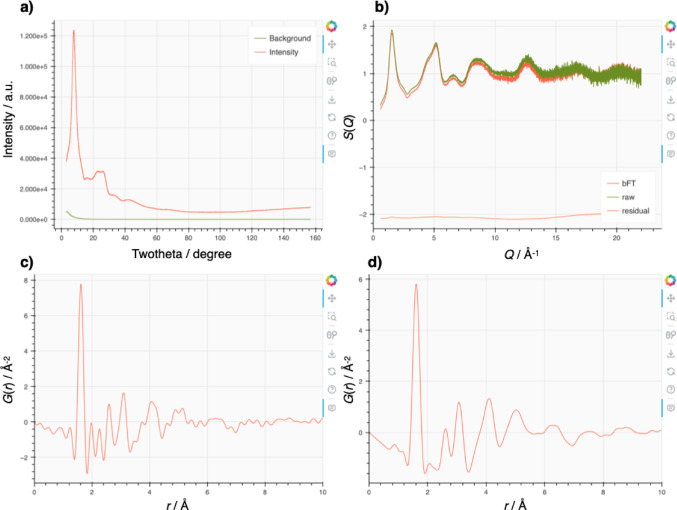
Typical results calculated using the WebPDF software. **a** X-ray total-scattering patterns of silica glass and the corresponding background profile obtained by Rigaku SmartLab diffractometer. **b** *S*(*Q*) profiles, including the experimental *S*(*Q*) (green), the back-Fourier-transformed profile *S*_bft(*Q*) (red), and the residual between them (orange). **c** *G*(*r*) obtained through Fourier transformation without applying a window function. **d** *G*(*r*) obtained using the Lorch window function. For clarity, axis labels have been overwritten from the original plots displayed in the software

Typical results obtained using the WebPDF software are presented in Fig. 1. As an example, a 0.5-mm-diameter SiO_2_ glass specimen was measured using a Rigaku SmartLab diffractometer equipped with a silver target (X-ray energy: 22.148 keV) [15]. Figure 1a shows the total-scattering patterns of both the specimen and the background capillary. These data allow evaluation of measurement quality, and when exposure times differ between sample and background, the background profile can be appropriately scaled prior to subtraction. From these inputs, the software generates *S*(*Q*) along with a normalized scattering profile and reference functions derived from the sum of atomic form factors and Compton scattering (Figures S1b–S1d). Because the experimental scattering intensity should oscillate around the theoretical reference curve (Figure S1c), parameters such as polarization and recoil factors—which affect the magnitude of Compton scattering—may require refinement. Proper adjustment of these parameters produces an *S*(*Q*) profile that oscillates around 1, as expected.

**Fig. 2:**
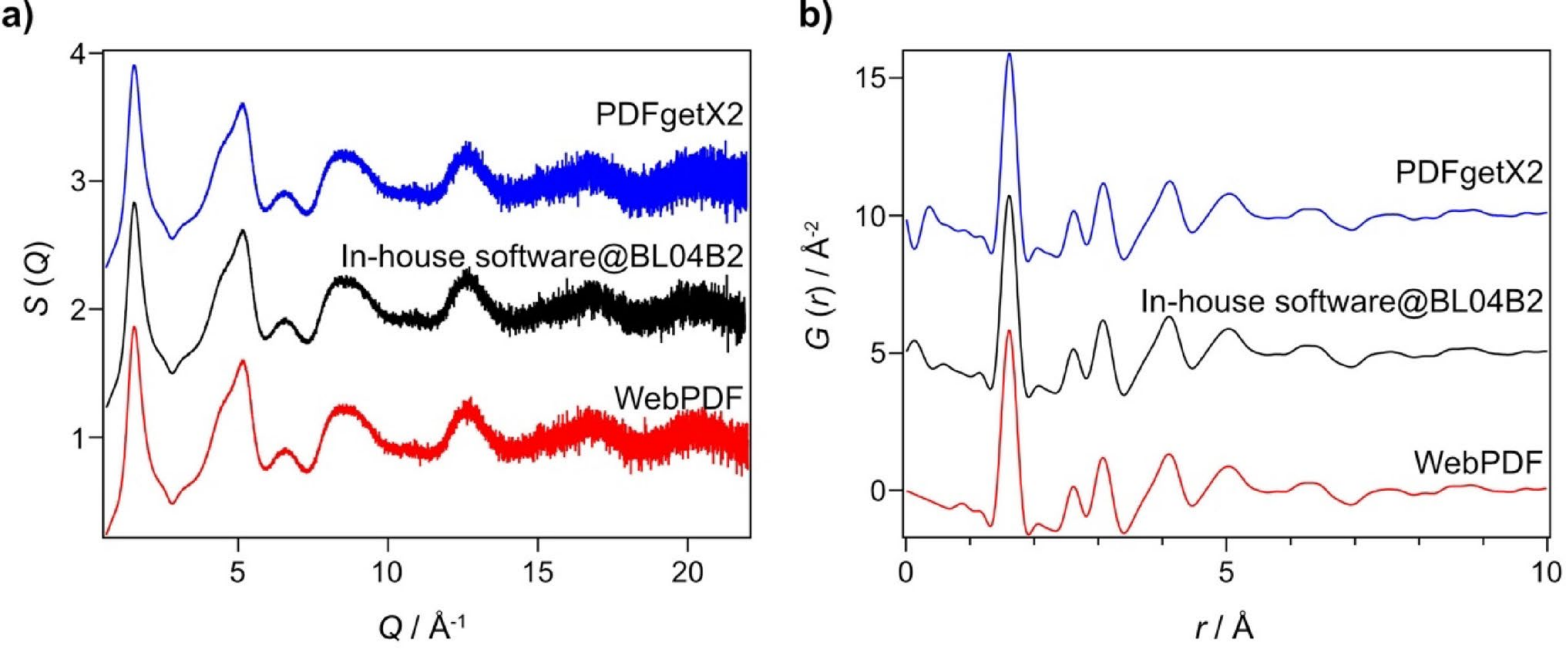
Comparison of *S*(*Q*) and *G*(*r*) for silica glass calculated by WebPDF, PDFgetX2, and the in-house software used at BL04B2 [22]. **a** *S*(*Q*). **b** *G*(*r*). The same X-ray scattering dataset measured using a Rigaku SmartLab diffractometer was used as input for all three software packages; only the data reduction and PDF calculation procedures differ

Subsequent Fourier transformation yields the PDF *G*(*r*). In this step, *S*(*Q*), the back-Fourier-transformed profile (${S}_{\mathrm{bft}}(Q)$), and their residuals are evaluated simultaneously (Fig. 1b). Minimizing the residual signal requires careful consideration of the low-*r* cutoff, the assumed atomic number density, and the polarization and recoil factors, all of which must remain physically reasonable. The program also outputs *G*(*r*) functions calculated with and without application of a Fourier-transform window function (Figs. 1c and d). The resulting profiles show good agreement with *S*(*Q*) functions and PDFs of SiO_2_ glasses generated using PDFgetX2 and the in-house software, which has been used for at least 15 years at BL04B2 in SPring-8 [22], confirming the reliability of the computational procedure (Fig. 2). Moreover, PDF analyses of real materials, such as Pt and PtCo nanoparticles used in fuel-cell applications, reproduce previously reported results [29], further demonstrating the robustness and accuracy of the software (Figure S2).

**Fig. 3:**
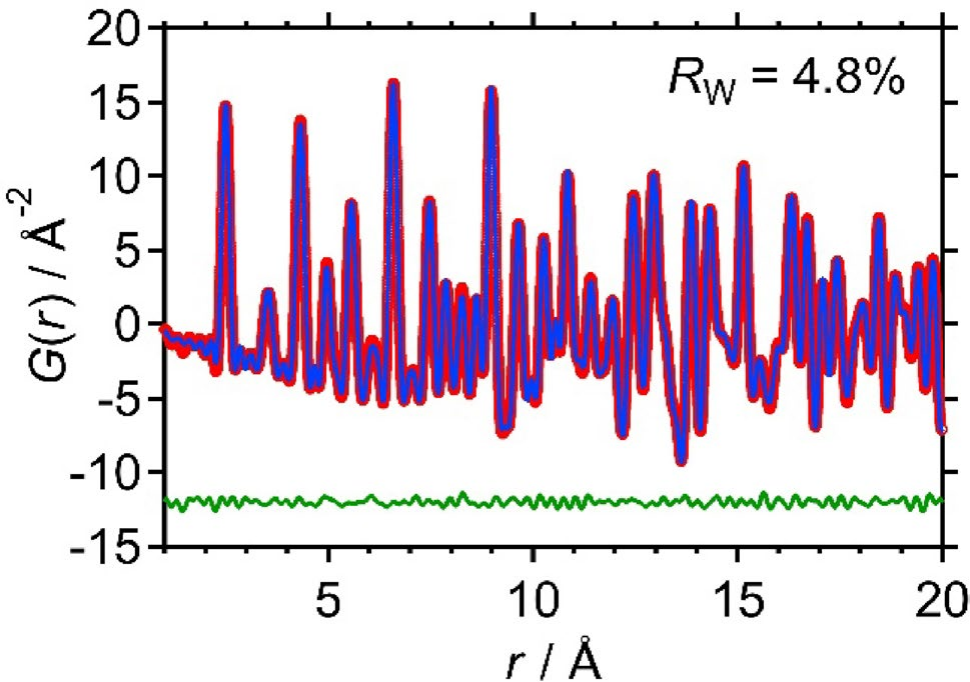
PDF fitting result for Ni. Red circles, blue lines, and green lines represent the experimental values, calculated values, and the difference curve, respectively. The fitting range of the Ni PDF pattern is 1–20 Å. The difference curve is vertically offset by − 13 for clarity

To validate the reliability of the PDF patterns generated by WebPDF, PDF refinement [30] was performed on the *G*(*r*) profile of Ni measured at BL04B2 (incident X-ray energy: 112.8 keV, Debye–Scherrer geometry, *Q*_max_ = 23 Å^−1^) under the same measurement conditions as in the previous study [8]. As shown in Fig. 3, the experimental and simulated profiles exhibit good agreement, yielding a weighted R-factor (*R*_w_) of 4.8%. This agreement confirms that the Fourier transformation and background-treatment procedures implemented in WebPDF are fully compatible with conventional refinement workflows.

**Fig. 4:**
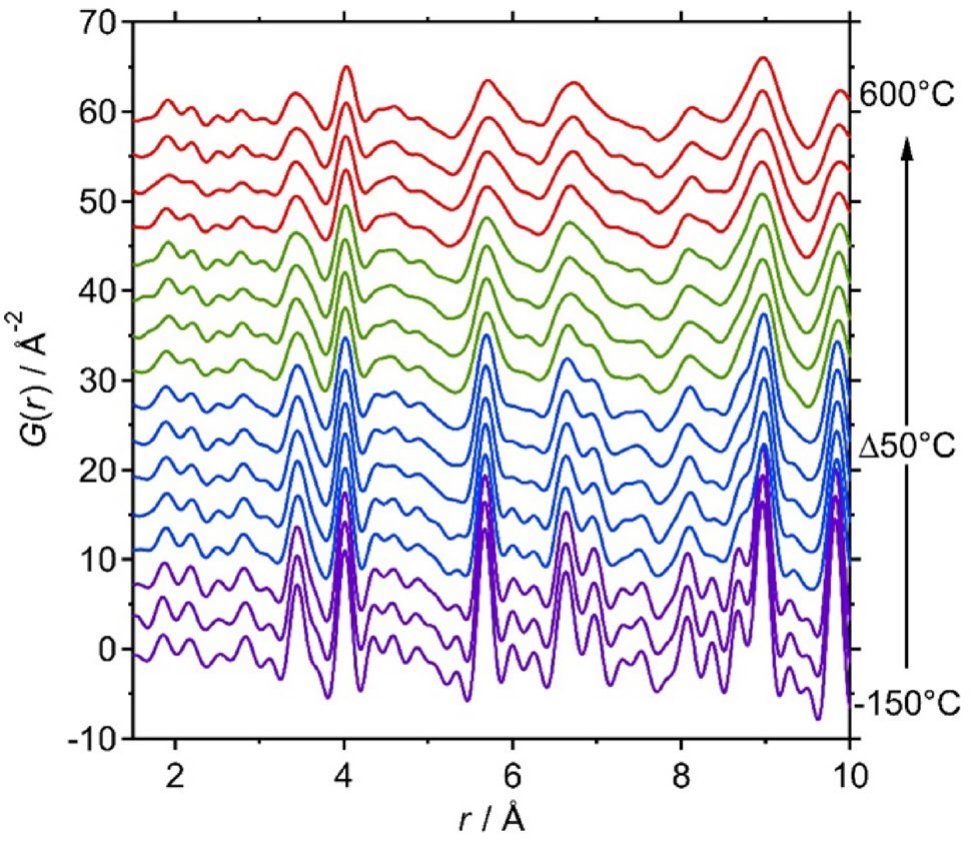
Temperature-dependent PDF patterns of KNbO_3_ obtained using sequential data reduction with the WebPDF software. Measurements were performed from − 150 °C to 600 °C in 50 °C increments. For clarity, the patterns are displayed with vertical offsets. The color–space group correspondence is as follows: red: *Pm* − 3* m*; green: *P4mm*; blue: *Amm2*; purple: *R3m*

An additional advantage of WebPDF is the computational efficiency achieved through optimized Python libraries such as NumPy and SciPy. The wall time for a typical calculation is approximately two orders of magnitude shorter than that of the in-house software at SPring-8, which is based on Igor Pro 8. This acceleration, combined with the browser-based execution environment and the assurance of fully local data processing, makes WebPDF a robust and practical tool for high-throughput PDF analysis. Multiple datasets can be uploaded and processed sequentially under a consistent set of parameters, enabling rapid comparison of structural evolution, such as phase transitions, crystallization behavior, or reaction mechanisms. Figure 4 presents an example of temperature-dependent PDF patterns for KNbO_3_ measured from − 150 °C to 600 °C (16 datasets). Using a typical laptop computer (Surface Pro 11, Intel Core Ultra 7-268 V), the X-ray scattering data covering 0.2°–24.002° in 2θ (step size: 0.006°, 3968 data points) were processed in approximately 16 s for all 16 datasets. This indicates that even datasets measured with fine angular resolution can be processed in approximately one second per dataset on a standard laptop. The data reveal pronounced structural changes in the 6–8 Å range, corresponding to the sequence of phase transitions in KNbO₃: cubic–tetragonal at ~ 435 °C, tetragonal–orthorhombic at ~ 225 °C, and orthorhombic–rhombohedral at − 10 °C, in agreement with previous X-ray diffraction studies [31]. As shown here, WebPDF can process large-scale sequential datasets comprising hundreds of files. Although such calculations naturally require longer wall times, they remain fully feasible within the browser environment, demonstrating the scalability of the software. This capability highlights the potential of WebPDF for applications involving extensive datasets, including operando measurements and automated high-throughput screening.

## Conclusion

A browser-based software, WebPDF, has been developed for calculating PDFs from X-ray total-scattering data. The program requires only minimal input files—sample and background patterns together with a JSON configuration file—and performs all computations locally in the browser using WebAssembly and PyScript. With NumPy and SciPy providing efficient numerical operations and Bokeh enabling interactive visualization, the software offers both high computational performance and user-friendly presentation of results. Representative analyses of SiO_2_ glass demonstrate that reliable *S*(*Q*) and *G*(*r*) functions can be obtained while reducing computation time by more than an order of magnitude compared with conventional implementations. The open-source release of WebPDF on GitHub promotes transparency, reproducibility, and community-driven development. Beyond its utility for high-throughput structural characterization, the browser-based design also makes the software well suited for educational use and rapid data inspection during synchrotron experiments. Future developments will focus on extending the framework to sequential and time-resolved PDF analyses, broadening its applicability to dynamic structural studies of disordered materials.

## Supplementary Information

Below is the link to the electronic supplementary material.

Supplementary Material 1

## Data Availability

All the original computer code using this software is open at the website (https://github.com/hirokiyamada1991/WebPDF/). The application of WebPDF and related data-reduction web-based software is also available at https://yamada-hiroki.com/software.html. All the experimental and simulated data used in this study are available from Hiroki Yamada on reasonable request.
