# Supplementary material for: WebPDF: a browser-based software application for calculating X-ray pair distribution function
Source: Anal Sci. 2026 May 1;42(8):719–26. doi: 10.1007/s44211-026-00917-x (PMC13400466; doi:10.1007/s44211-026-00917-x)
Supplement: Supplementary file 1 — Supplementary Material 1 [file 44211_2026_917_MOESM1_ESM.docx]

**Fig. S1** Structure factor and related profiles of silica glass obtained using WebPDF software. (a) X-ray total-scattering patterns of silica glass and the corresponding background profile. (b) *S*_raw(*Q*). (c) Normalized scattering intensity alongside the profile of the sum of atomic form factors and Compton scattering. (d) Normalized scattering intensity with individual contributions from atomic form factors and Compton scattering. Axis labels have been overwritten for clarity


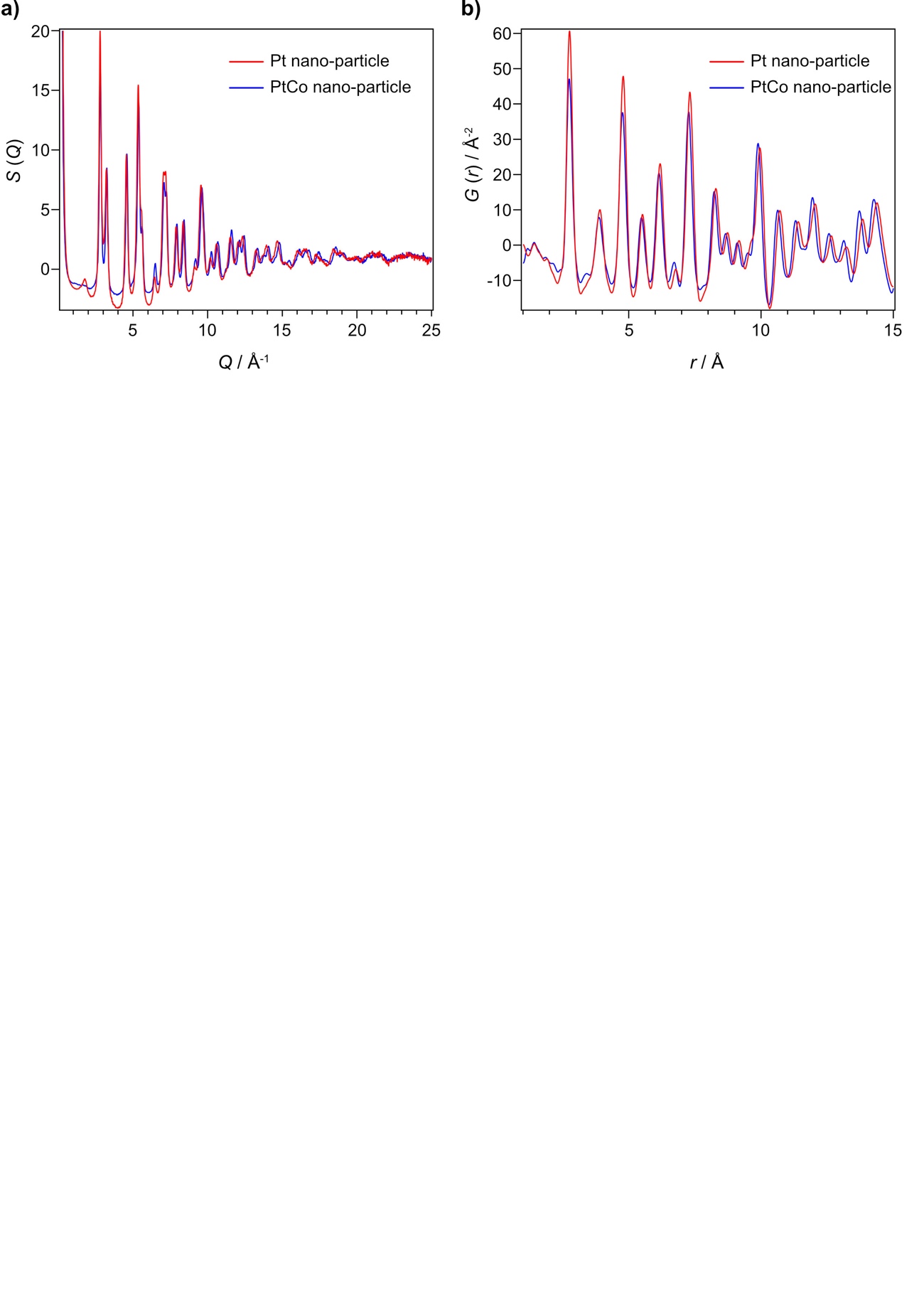


**Fig. S2** *S*(*Q*) and *G*(*r*) profiles of Pt and PtCo nanoparticles obtained using WebPDF software
